# Efficacy and safety of Yinaokang capsules on functional recovery after acute ischemic stroke: protocol for a pilot, prospective, randomized, open-label, blinded-endpoint trial

**DOI:** 10.3389/fneur.2026.1888277

**Published:** 2026-08-26

**Authors:** Yi-Hang Li, Yu-Hui Qiu, He-Jin Wang, Min-Hua Zheng, Ling-Ling Liu, Shi-Jie Zhang, Ye-Feng Cai

**Affiliations:** 1 Department of Neurology, The Second Affiliated Hospital of Guangzhou University of Chinese Medicine, Guangzhou, Guangdong, China; 2 Department of Neurology, Guangdong Provincial Hospital of Chinese Medicine, Guangzhou, Guangdong, China; 3 Science and Technology Innovation Center, Guangzhou University of Chinese Medicine, Guangzhou, Guangdong, China

**Keywords:** acute ischemic stroke, protocol, randomized controlled trial, traditional Chinese medicine, Yinaokang capsules

## Abstract

**Background:**

Previous studies suggest that Yinaokang Capsules (YNKC) may offer therapeutic benefits for acute ischemic stroke (AIS) by reducing atherosclerosis, inhibiting inflammatory mediators, and exhibiting neuroprotective properties. Nonetheless, there is currently a lack of substantial clinical data that validates the overall efficacy and safety of YNKC. Furthermore, the systemic metabolic changes that underpin its neurovascular protective effects have not been thoroughly investigated in clinical contexts.

**Methods:**

This study is a prospective, randomized, open-label, blinded-endpoint trial designed to enroll 80 patients diagnosed with AIS aged between 18 and 80 years at the Guangdong Provincial Hospital of Chinese Medicine. Eligible participants must present within 72 h of symptom onset and have a baseline National Institutes of Health Stroke Scale (NIHSS) score ranging from 4 to 22. Participants will be randomly assigned in a 1:1 ratio to either the YNKC group or the control group. Those in the YNKC group will receive four YNKC orally three times daily for a duration of 14 days. All participants will concurrently receive standard guideline-based medical treatment. The primary efficacy outcome will be the proportion of patients achieving a favorable functional outcome, defined as a modified Rankin Scale (mRS) score of 0 to 1 at day 90 following randomization. Secondary outcomes included the overall distribution of mRS scores, NIHSS score, Montreal Cognitive Assessment (MoCA) scores, Traditional Chinese Medicine (TCM) Syndrome Scores, Barthel Index (BI) score, the Stroke-Specific Quality of Life (SS-QoL) scores. The main safety outcome will be the incidence of adverse events (AEs) and serious adverse events (SAEs). Additionally, targeted blood metabolomics will be conducted to explore the underlying metabolic mechanisms of YNKC.

**Conclusion:**

Aims to evaluate the impact of YNKC on day 90 functional prognosis of patients with AIS, and to explore its underlying metabolic mechanisms.

**Clinical trial registration:**

itmctr.ccebtcm.org.cn, ITMCTR2025001037.

## Introduction

Stroke continues to be a leading cause of mortality and long-term disability globally (1, 2), with China exhibiting the highest estimated lifetime risk in the world (3). In 2020, China reported approximately 17.8 million prevalent cases, 3.4 million incident cases, and 2.3 million stroke-related deaths (4). This substantial disease burden has resulted in the highest economic costs associated with stroke in China (5).

Ischemic stroke constitutes the majority of these cases, accounting for 70–83% (6). Although reperfusion therapies for acute ischemic stroke (AIS) have made significant advancements in recent decades, their clinical application is still severely limited by narrow eligibility criteria. Recent national data indicate that the overall rates of intravenous thrombolysis and endovascular treatment in China are only 5.64 and 1.45%, respectively (7). Given that most AIS patients do not qualify for these standard reperfusion therapies, there is an urgent need for widely accessible alternative treatments. As a result, Traditional Chinese Medicine (TCM) has gained increasing attention as a promising therapeutic strategy (8, 9).

The Yinaokang Capsules (YNKC) comprises eight traditional Chinese medicinal ingredients: Huangqi (Astragali Radix), Chuanxiong (Chuanxiong Rhizoma), Zhi Tianma (Processed Gastrodiae Rhizoma), Zhi Nanxing (Processed Arisaematis Rhizoma), Yimucao (Leonuri Herba), Zhi Banxia (Processed Pinelliae Rhizoma), Shichangpu (Acori Tatarinowii Rhizoma), and Quanxie (Scorpio). This formulation is produced by the Guangdong Provincial Hospital of Traditional Chinese Medicine and has been approved by the National Medical Products Administration (Approval No.: Z20071026). To ensure the quality consistency and batch-to-batch stability of Yinaokang preparations, we have systematically established high-performance liquid chromatography fingerprint chromatograms and multi-index component content determination methods for Yinaokang extracts in the early stage. The content determination results show that the characteristic compounds with clear pharmacological activity in the 10 batches of samples meet the requirements of the Chinese Pharmacopoeia and the FDA’s guidelines for the validation of bioanalytical methods for quantitative analysis methods (10). Preliminary studies indicate that this formulation not only corrects endothelial dysfunction, improves ischemic–hypoxic brain injury, alleviates cerebral edema, and mitigates blood–brain barrier disruption following thrombolysis, but it may also regulate oxidative stress and cellular function (11). Early clinical evidence suggests that a comprehensive traditional Chinese medicine approach, which includes YNKC, can enhance therapeutic efficacy and quality of life in patients with AIS (12). Despite the promising clinical outcomes, the specific systemic metabolic mechanisms that underlie the neuroprotective effects of YNKC remain inadequately understood. AIS induces significant metabolic reprogramming, which is marked by severe energy crises and cascades of oxidative stress. To address this gap, this study will employ a targeted blood metabolomics approach. By profiling systemic metabolic changes, we aim to clarify how YNKC influences the ischemic metabolic microenvironment. However, before proceeding to large-scale confirmatory trials, the preliminary efficacy and safety of this formulation require systematic evaluation. Consequently, this prospective, randomized, open-label, blinded-endpoint trial is designed as an exploratory pilot study. It seeks to preliminarily assess the impact of YNKC on the 90 days functional prognosis of patients with AIS, evaluate its safety profile, and to uncover its underlying metabolic regulatory mechanisms, thereby providing critical feasibility data and effect size estimates for future, adequately powered Phase III trials.

## Methods and design

The protocol has been developed and presented in accordance with the Standard Protocol Items: Recommendations for Interventional Trials (13). The schedule of enrollment, intervention, and assessment is provided in Table 1.

**Table 1:** SPIRIT schedule of enrollment, intervention, and assessment.

Time point	STUDY PERIOD					
	Enrollment	Allocation	Post-allocation	Follow-up		
	Pre-randomization	after evaluation	Baseline	Visit 1 (Day 7)	Visit 2 (Day 14 ± 2)	Visit 3 (Day 90 ± 7)
Enrollment						
Inclusion/Excluson criteria	×		×			
Informed consent	×		×			
Randomization		×				
Demographics	×					
Medical history	×		×			
Interventions						
YNK Capsule group			×			
Control group			×			
Assessments						
Physical examination			×			×
Vital signs			×	×	×	×
12-lead ECG			×		×	
CT/MRI			×			
Laboratory tests (Blood/Urine)			×	×		
NIHSS score			×		×	×
TCM Syndrome Scale			×		×	×
mRS score			×			×
BI						×
MoCA score					×	×
SS-QoL scale						×
Adverse events			×	×	×	×
Concomitant medications			×	×	×	
Medication adherence				×	×	

CT, computed tomography; MRI, magnetic resonance imaging; ECG, electrocardiogram; NIHSS, National Institutes of Health Stroke Scale; TCM, Traditional Chinese Medicine; mRS, modified Rankin Scale; BI, Barthel Index; MoCA, Montreal Cognitive Assessment; SS-QoL, Stroke Specific Quality of Life Scale; AEs, Adverse events.

### Trial design

**Figure 1:**
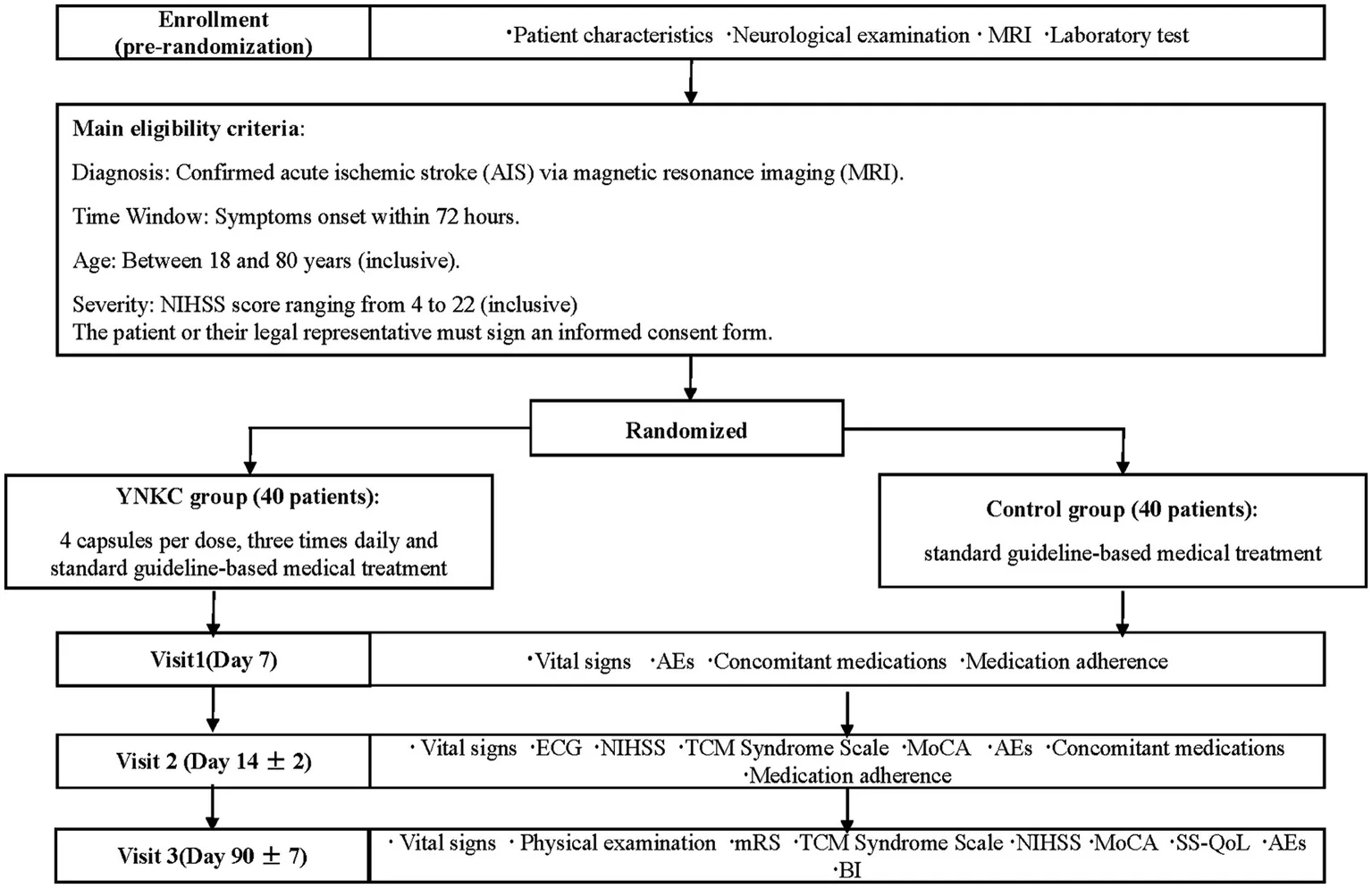
Study flowchart. YNKC, Yinaokang Capsules; CT, computed tomography; MRI, magnetic resonance imaging; ECG, electrocardiogram; NIHSS, National Institutes of Health Stroke Scale; TCM, Traditional Chinese Medicine; mRS, modified Rankin Scale; BI, Barthel Index; MoCA, Montreal Cognitive Assessment; SS-QoL, Stroke Specific Quality of Life Scale; PRO, patient-reported outcomes; AEs, adverse events. License:© 2025 Chan A-W et al. (13). This is an Open Access article distributed under the terms of the Creative Commons Attribution License (https://creativecommons.org/licenses/by/4.0/), which permits unrestricted use, distribution, and reproduction in any medium, provided the original work is properly cited.Source: https://dx.doi.org/10.1136/bmj-2024-081477

A prospective, randomized, open-label, blinded-endpoint trial aims to evaluate the effectiveness and safety of YNKC in promoting functional recovery following AIS. Patient recruitment is planned at the Guangdong Provincial Hospital of Chinese Medicine from June 2025 to October 2026. The study received approval from the Ethics Committee of Guangdong Provincial Hospital of Chinese Medicine (BF2025-006-01), in accordance with the criteria established by the Helsinki Declaration. Additionally, it was registered on itmctr.ccebtcm.org.cn (ITMCTR2025001037) on May 26, 2025. Trained clinicians within the study team will inform patients about the study’s objectives, risks, and benefits. Written informed consent will be obtained from participants or their legal surrogates. A flowchart summarizing the study design is presented in Figure 1.

### Study population

#### Inclusion criteria

Patients must meet all of the following criteria to be eligible for enrollment:

1. Diagnosis: Confirmed AIS via magnetic resonance imaging (MRI) (14).
2. Time window: Symptoms onset within 72 h.
3. Age: Between 18 and 80 years (inclusive).
4. Severity: National Institutes of Health Stroke Scale (NIHSS) score ranging from 4 to 22 (inclusive).
5. Traditional Chinese Medicine Syndrome (Phlegm Syndrome): Meets the diagnostic criteria for phlegm-dampness syndrome, as indicated by a score of ≥10 on the Ischemic Stroke Syndrome Element Diagnostic Scale (15).
6. Traditional Chinese Medicine Syndrome (Blood Stasis Syndrome): Meets the diagnostic criteria for blood stasis syndrome, as indicated by a score of ≥10 on the Ischemic Stroke Syndrome Element Diagnostic Scale (15).
7. Informed consent: The patient or their legal representative must sign an informed consent form.

Patients meeting any of the following criteria will be excluded from the study:

1. Stroke of cardioembolic etiology.
2. Receipt or scheduling of intravenous thrombolysis, endovascular treatment (including mechanical thrombectomy, intra-arterial thrombolysis, and angioplasty), or anticipation of endovascular or surgical interventions within the next 3 months.
3. Use of other TCM decoctions/granules or Chinese patent medicines for stroke treatment post-onset of the current episode.
4. Stroke resulting from non-vascular causes such as brain tumor, brain trauma, hematologic diseases, infectious diseases, hereditary diseases, or rheumatic immune diseases.
5. History of stroke with residual sequelae impacting outcome assessment, with a pre-stroke mRS score ≥ 2 (16).
6. Co-morbidities leading to physical disability that hinders neurological assessment (e.g., claudication, osteoarthritis, rheumatoid arthritis, or gouty arthritis).
7. Severe hepatic or renal dysfunction. Hepatic dysfunction is defined as ALanine aminoTransferase (ALT) or ASpartate aminoTransferase (AST) > 2 times the upper limit of normal (ULN), while renal dysfunction is defined as serum creatinine > 2 times the ULN.
8. Life expectancy: Presence of other life-threatening conditions with a life expectancy of less than 3 months.
9. Evaluation barriers: Any condition that limits neurological evaluation or precludes patient follow-up.
10. Pregnancy/Lactation: Women who are pregnant, planning to become pregnant, or breastfeeding.
11. Other trials: Current participation in other interventional clinical trials.
12. Severity/Administration issues: Severe disturbance of consciousness (NIHSS item 1a greater than 1); dysphagia (difficulty swallowing) preventing oral administration of the YNKC.
13. Hematologic/Coagulation disorders: Coagulation disorders, systemic bleeding, leukopenia (less than 2 × 10^9^/L), or thrombocytopenia (less than 100 × 10^9^/L).
14. Allergy: Known history of hypersensitivity to the YNKC or any of its components.
15. Active ulcer: Active peptic ulcer disease.
16. Bleeding/Surgery history: History of gastrointestinal bleeding within 3 months prior to randomization, or major surgery within 30 days prior to randomization.

### Sample size calculation

This single-center, prospective exploratory pilot trial does not aim to confirm efficacy. Its primary goals are to evaluate the feasibility of the YNKC regimen in patients with acute ischemic stroke—specifically enrollment rate, treatment adherence, and operational workflow—while also screening for preliminary safety signals and generating key parameters for a later phase III confirmatory trial (17).

Following common practice in stroke pilot studies, the total sample size was set at 80 participants (experimental: control = 1:1). This scale aligns with conventions for exploratory studies and is adequate to: (1) preliminarily assess between-group trends and the variability of functional outcomes (for example, mRS); (2) estimate the standard deviations and event rates needed for future confirmatory testing; and (3) identify potential safety signals and procedural bottlenecks (18). It must be emphasized that pilot studies commonly overestimate effect sizes (17). Results from this trial will be used only for parameter estimation and feasibility assessment and not as evidence of efficacy. The sample size for the subsequent confirmatory trial will be determined using the variance and event-rate estimates obtained here, balanced against the minimum clinically important difference, and may be adjusted via adaptive methods (for example, sample-size re-estimation).

### Randomization

Randomization was performed by an independent statistician who was not involved in participant recruitment, intervention delivery, or outcome assessment. Prior to enrollment, a centralized, web-based randomization system was established. Participants were randomized in a 1:1 ratio to either the YNKC group or the control group using minimization with a random component, a validated covariate-adaptive randomization method recommended for small-to-moderate-sized acute stroke trials (19). The algorithm balanced key prognostic baseline covariates known to influence stroke outcomes, including: (1) age (<70 vs. ≥ 70 years), (2) baseline National Institutes of Health Stroke Scale (NIHSS) score (≤5 vs. > 5), (3) stroke subtype (large-artery atherosclerosis vs. non-large-artery), and (4) time from symptom onset to randomization (<48 h vs. ≥ 48 h). At each new enrollment, the system calculated the current imbalance across these factors between the two groups and assigned the participant to the group that minimized overall imbalance, with a pre-specified probability (e.g., 0.8) favoring balance and 0.2 introducing randomness to preserve allocation unpredictability. Sequential study identification numbers (1–80) were assigned based on enrollment order, and treatment allocation was concealed until after eligibility confirmation.

### Interventions

Following confirmation of eligibility, participants will be randomly assigned in a 1:1 ratio to either the YNKC group or the control group. Those in the YNKC group will receive oral YNKC (4 capsules per dose, three times daily) in conjunction with standard guideline-based medical treatment, which primarily consists of conservative pharmacological interventions, including antiplatelet, anticoagulant, and defibrinogenation therapies, as well as routine management of vascular risk factors (14). In contrast, the control group will receive only the standard guideline-based treatment. The duration of the treatment course for both groups will be 14 days.

### Management of investigational products

The drug administrator is responsible for the storage of the experimental drug. The drug administrator must be authorized by the principal investigator. When the sponsor transfers the experimental drug to the center, the drug managers must verify the condition of the drug. They must sign and date the drug handover form and include batch number, quantity and expiration date. Once the drug has been received, the drug managers must store it securely and according to the storage requirements. Both the investigator and the drug manager have to ensure that the experimental drug is given only to the subjects in the study. The drug administrator must accurately and promptly record the distribution and recall of the experimental drug in the distribution and recall forms. After the clinical trial, any remaining drug must be withdrawn. In accordance with the required requirements, both the drug administrator and the sponsor supervisor will sign the drug recall handover form.

### Criteria for discontinuing or modifying allocated intervention

1 Modification of allocated intervention: The fixed-dose regimen of the YNKC typically does not allow for dose reduction, such as halving the dose. However, if mild or moderate adverse events, such as gastrointestinal intolerance, arise and are suspected to be related to the study drug, the Principal Investigator may use clinical judgment to temporarily interrupt the intervention. This interruption can last for a maximum of 3 consecutive days.

i If the symptoms resolve or improve to an acceptable level during this period, the study medication may be resumed at the original dose.
ii If the symptoms persist or worsen despite the temporary interruption, the intervention must be permanently discontinued.

2 Criteria for permanent discontinuation: The intervention allocated to the YNKC group must be permanently discontinued for a participant if any of the following criteria are met:

i Safety and tolerability:
• The occurrence of a Serious Adverse Event (SAE) or a Suspected Unexpected Serious Adverse Reaction (SUSAR) that, in the investigator’s opinion, compromises the participant’s safety.
• The presence of severe hypersensitivity or allergic reactions to the investigational product.
• The identification of significant abnormalities in laboratory parameters (e.g., ALT/AST > 3x Upper Limit of Normal or a significant worsening of renal function) that necessitate the cessation of the intervention.
ii Disease progression and clinical status:
• Hemorrhagic transformation of cerebral infarction confirmed through neuroimaging, either by Computed Tomography (CT) or MRI.
• Clinical deterioration that prevents the administration of medication orally or via nasogastric tube, such as the onset of severe dysphagia or coma.
• The necessity for emergency surgery or the presence of prohibited concomitant medications that may interact with the study drug.
iii Participant request:

• Withdrawal of consent for continued treatment by the participant or their legal surrogate. Note that this is distinct from the withdrawal of consent for study follow-up.
3 Follow-up for participants who discontinue intervention discontinuation of the investigational product does not equate to withdrawal from the study. To minimize missing data and facilitate Intention-to-Treat (ITT) analysis:
i Participants who discontinue the study medication will be encouraged to remain in the trial. They will continue to undergo all scheduled assessments, including NIHSS and mRS evaluations, at the protocol-defined time points (e.g., Day 14, Day 90), unless they explicitly withdraw their consent for any further data collection.

### Concomitant medications

#### Permitted concomitant medications/treatments

Standard Western medical treatments relevant to the patient’s condition may be administered in accordance with current clinical guidelines. Permitted medications include mannitol, glycerol fructose, diuretics, normal saline, glucose, as well as antihypertensive, hypoglycemic, and lipid-lowering agents. The use of neuroprotective agents, such as edaravone and vinpocetine, is also allowed.

#### Prohibited concomitant medications/treatments

During the study treatment period, the use of TCM injections or proprietary Chinese medicines (patent medicines) indicated for stroke is strictly prohibited. Specific examples of prohibited medications include Sodium Aescinate (Aescin), Ligustrazine (Chuanxiong), and Erigeron breviscapus (Dengzhanxixin).

### Data collection and management

Investigators are tasked with the timely, accurate, complete, and legible transcription of data from source documents into the electronic Case Report Form (eCRF). It is essential that the eCRF is completed promptly for each enrolled participant. The data collection instruments comprise patient questionnaires, eCRFs, and telephone interview questionnaires. All data collection forms can be obtained from the corresponding author upon request.

### Participant retention strategy

A multifaceted approach will be employed to enhance participant retention. A patient-centered strategy will be maintained throughout all trial activities and interactions. This will involve engaging participants during the trial’s development, initiation, and implementation phases, primarily through consultations with the Consumer Advisory Board. Investigators will be readily available to address any inquiries or concerns from participants at all times. To reduce the burden on participants’ time, study visits will be scheduled to align with their existing outpatient or other medical appointments whenever possible. For study-specific visits that cannot be conducted remotely (e.g., via telephone), parking vouchers or transportation subsidies, will be provided. For participants who choose to withdraw from the trial, no further data will be collected from the date of withdrawal.

### Data management

Data Verification Before the initiation of the study, a Data Validation Plan (DVP) will be collaboratively developed by the investigators, statisticians, clinical research monitors, and data managers. Data personnel will review the data in accordance with the DVP, focusing on identifying missing values, logical errors, and inconsistencies. Any discrepancies found will be documented in Data Query Forms (DQFs) and returned to the investigators for clarification and resolution. Once the queries are resolved, the data manager will update the database and prepare a Data Review Report. The Data Manager will refine the database based on the Data Validation Report and proceed with the database lock. This locking process must be conducted jointly by the Principal Investigator’s Unit and the statisticians. In principle, no further modifications will be permitted to the locked data files. Following this, the Data Manager will export and back up the locked database. The locked database, along with documents generated during the data management process—such as laboratory reference ranges and DQFs—will be formally handed over to the statisticians through a written procedure.

### Confidentiality

All collected materials will be utilized solely for research purposes, ensuring strict confidentiality throughout the process. Personally identifiable information (PII) will not be disclosed to any third party without the explicit consent of the subject. Data will be de-identified and stored under unique study identification numbers assigned to each participant. Access to this data will be limited to authorized personnel involved in the study. Physical records will be securely stored in locked facilities, while electronic data will be protected through password encryption. Individual participants will remain unidentified in any reports, presentations, or publications that arise from this study.

### Sample collection and laboratory analysis

Day 7 marks the subacute phase after stroke. By this time, the initial wave of cell necrosis has begun to subside, while secondary injuries—such as oxidative stress, neuroinflammation, and metabolic reprogramming—remain active. This period is therefore critical for assessing how interventions regulate metabolic pathways. Moreover, disruptions in tricarboxylic acid (TCA) cycle intermediates not only indicate mitochondrial dysfunction but are also tightly linked to inflammatory activation and oxidative stress (20). These processes remain active on the 7th day, so detecting the plasma metabolic profile at this time can effectively capture the regulatory effect of the intervention on these core pathological pathways.

Fasting venous blood samples were collected on Day 7 of treatment to assess early metabolic adaptations during the active intervention phase. Blood was drawn into EDTA tubes and centrifuged at 4 °C within 30 min. The plasma was then aliquoted and stored at −80 °C. Targeted metabolomics profiling was conducted using LC–MS/MS, which quantified 32 key metabolites involved in glycolysis (e.g., glucose-6-phosphate, pyruvate), the TCA cycle (citrate, *α*-ketoglutarate, succinate), and oxidative stress pathways (GSH/GSSG, 8-OHdG). This analysis followed established protocols for central carbon metabolism. Quality control (QC) samples were created by pooling equal volumes of all plasma samples. These QC samples were injected at the beginning, every eight samples, and at the end of each analytical batch to monitor system stability and correct for instrumental drift (20). Features with a coefficient of variation (CV) greater than 30% were excluded from the analysis. The raw data underwent normalization using Probabilistic Quotient Normalization to mitigate the effects of sample dilution. Principal Component Analysis was employed to evaluate batch effects. If batch effects were identified, either ComBat or LOESS correction would be implemented.

### Trial outcomes

The primary outcome is the proportion of patients achieving a favorable functional outcome, defined as a mRS score of 0 to 1, at day 90 (± 7). The mRS is a commonly utilized tool for assessing functional recovery following a stroke. It is coded on a scale from 0, indicating no symptoms, to 5, representing severe disability, and 6, denoting death. A score ranging from 0 to 1 signifies an excellent outcome, indicating that the individual is disability-free. Secondary outcomes included several key measures. To thoroughly assess clinically significant improvements among patients with varying severity levels, we have broadened the key secondary outcomes to include the following: the overall distribution of mRS scores (0–6) at day 90, analyzed through an ordinal shift analysis, and the proportion of patients attaining functional independence, defined as a mRS score of 0–2 at day 90. Neurological improvement is assessed by the proportion of patients exhibiting early neurological improvement, defined as either an NIHSS score of ≤ 1 or a reduction in NIHSS score of ≥ 4 from baseline, at Day 14 (± 2) and Day 90 (± 7). Cognitive function is evaluated using Montreal Cognitive Assessment (MoCA) scores at Day 14 (± 2) and Day 90 (± 7). TCM efficacy was determined by changes in TCM Syndrome Scores at Day 14 (± 2) and Day 90 (± 7). Activities of daily living (ADL) are measured by the proportion of patients achieving functional independence, defined as a Barthel Index (BI) score of ≥ 85, at Day 90 (± 7). Quality of life was assessed using the Stroke-Specific Quality of Life (SS-QoL) scores at Day 90 (± 7). Additionally, the incidence of symptomatic ischemic stroke during the follow-up period is recorded to evaluate the recurrence of stroke. Finally, the incidence of composite cardiovascular and cerebrovascular events during the follow-up period is also documented as part of vascular events.

The treatment allocation will be disclosed to both investigators and participants, resulting in an open-label design that reflects real-world clinical practice. To address potential ascertainment bias and maintain consistency, the study implements a blinded endpoint assessment strategy. All primary and secondary clinical outcomes—including the Day 90 mRS score, NIHSS, MoCA, BI score, SS-QoL, and TCM syndrome scores—will be evaluated by trained clinical assessors who are strictly blinded to the treatment assignments. To rigorously maintain this blind and minimize assessment bias within the PROBE design, several practical measures will be implemented.

First, the outcome assessors will be completely independent from the treating physicians; they will not participate in ward rounds, clinical decision-making, or the administration of the YNKC intervention. Second, prior to each scheduled evaluation, a standard script will be read to the participants and their authorized caregivers, explicitly instructing and reminding them not to disclose any information regarding their specific treatment allocation or medications to the evaluating staff.

Furthermore, a predefined protocol is established to manage accidental unblinding. In the event that a participant inadvertently reveals their group assignment, or if an assessor becomes unblinded for any other reason, the incident will be immediately documented in the Trial Master File. The compromised assessor will be recused from evaluating that specific patient, and a designated, fully blinded backup assessor will be promptly assigned to conduct all subsequent assessments for that individual, thereby preserving the integrity of the endpoint evaluations.

### Safety assessment

#### The primary safety outcomes

Incidence of AEs and SAEs at 90 Days, as reported by the investigators. AEs are defined as any unfavorable medical occurrence in a patient or clinical investigation subject who has received a pharmaceutical product, regardless of whether there is a causal relationship with the treatment. These events will be recorded throughout the treatment and follow-up periods. AEs in this study will be standardized and graded according to version 5.0 of the Common Terminology Criteria for Adverse Events (CTCAE v5.0) from the U. S. National Cancer Institute. CTCAE v5.0 defines five severity levels:

- Level 1 (mild): Asymptomatic or mild symptoms; only clinical or diagnostic findings; no intervention required.
- Grade 2 (moderate): Requires minimal, local, or noninvasive intervention; limits instrumental activities of daily living (e.g., shopping, housework).
- Grade 3 (severe or medically significant but not immediately life-threatening): Requires hospitalization or prolongation of hospitalization; results in disability; limits self-care activities of daily living (e.g., bathing, dressing).
- Grade 4 (life-threatening): Requires urgent intervention to prevent death.
- Grade 5 (death): Death related to the AE.

#### The second safety outcomes

##### Laboratory parameters

This includes the assessment of absolute neutrophil count, platelet count, liver and renal function tests, and coagulation profiles.

### Exploratory outcomes

Alongside the clinical evaluations, this study incorporates a targeted blood metabolomics analysis as an exploratory outcome. By correlating dynamic metabolic changes with clinical functional outcomes, this sub-study seeks to delineate the multi-target regulatory network of YNKC involved in the repair of the ischemic metabolic microenvironment.

### Adverse events management

All AEs, regardless of whether they are reported by the subject, observed by the investigator, or identified through physical examinations, laboratory tests, or other methods, must be documented in the AE section of the CRF. These events will be managed in strict compliance with applicable regulations. Affected patients will receive appropriate medical management, and the outcomes will be meticulously recorded. Follow-up will persist until the event either resolves or stabilizes. The decision to discontinue YNKC will be assessed by the principal investigator in collaboration with a neurologist. This evaluation will be based on predefined medical judgment criteria. Specifically, discontinuation will be considered if any of the following situations arise, clearly attributed to the study intervention, as defined by the WHO-UMC causality assessment criteria: (a) Any grade 3 or higher bleeding events, such as intracranial hemorrhage or massive gastrointestinal hemorrhage; (b) Allergic reactions or angioedema, particularly those affecting the respiratory tract; (c) New-onset status epilepticus or uncontrollable severe arrhythmia; (d) Significantly abnormal liver function, defined as ALT/AST levels greater than five times the upper limit of normal (ULN), accompanied by elevated bilirubin levels exceeding two times the ULN, in accordance with Hy’s rule; (e) Other SAEs deemed unacceptable risks by the researcher, even if the causal relationship remains uncertain, as continued intervention may jeopardize the safety of the subjects. If any of the aforementioned conditions are met, the research intervention will be immediately and permanently discontinued. Subsequently, standard medical treatment will be initiated. Despite this discontinuation, the subject will remain included in the final efficacy and safety analysis, adhering to the principle of ITT analysis to mitigate bias. Furthermore, all serious adverse events (SAEs) will be reported to the institutional ethics committee within 24 h of their occurrence, and a comprehensive follow-up report will be submitted within 7 days. The Data Security Monitoring Committee will regularly review the accumulated safety data, specifically after every 20 cases included, and will recommend suspension or termination of the trial if deemed necessary.

If any of the above conditions occurs, the research intervention will be stopped immediately and permanently, and standard medical treatment will begin. The subject will nevertheless remain in the final efficacy and safety analyses under the ITT principle to avoid bias. All SAEs will be reported to the institutional ethics committee within 24 h, with a detailed follow-up report submitted within 7 days. The Data Safety Monitoring Committee will review accumulated safety data regularly (every 20 enrolled cases) and may recommend suspension or termination of the trial if warranted.

### Data monitoring body

This study is a single-center, open-label investigation conducted at the Guangdong Provincial Hospital of Chinese Medicine. The principal investigator’s team will directly manage the study, thereby eliminating the need for a separate administrative or data coordination center. Participant recruitment and daily operations will take place within the hospital under the principal investigator’s direct supervision. To ensure scientific rigor and objectivity, an independent Data Monitoring and Safety Committee (DMSC) will be established. This committee will convene biannually to oversee the study’s progress, with a focus on patient safety, adherence to the protocol, and the integrity of the data. In terms of data management, designated data managers within the research team will be responsible for data cleaning and quality control using the eCRF system, rather than relying on an external data center. Regular data quality reports will be generated and reviewed collaboratively by the principal investigator and the DMSC to maintain the study’s validity and credibility.

### Statistical analysis

The statistical analysis will be performed using R software (version 4.4.3). A two-sided *p*-value of <0.05 will be considered statistically significant.

### Analysis sets

Efficacy analyses will primarily focus on the ITT population, which includes all randomized subjects. These analyses will be further supported by the Per-Protocol (PP) population, consisting of subjects who completed the protocol without significant deviations. Safety analyses will be conducted on the Safety Set (SS), defined as all randomized patients who received at least one dose of the study medication.

### General statistical principles

Descriptive statistics were employed to summarize and compare baseline characteristics among the enrolled patients, ensuring inter-group comparability. Categorical variables were reported as frequencies and percentages (*n*, %), and comparisons were made using Pearson’s chi-squared test or Fisher’s exact test when any expected cell count was less than 5. Prior to analysis, continuous variables were assessed for normal distribution. Normally distributed continuous variables were expressed as mean ± standard deviation (SD). For these variables, inter-group comparisons were performed using the independent-samples *t*-test for two groups or one-way analysis of variance (ANOVA) for multiple groups. Intra-group changes (pre- vs. post-treatment) were evaluated with the paired *t*-test. Non-normally distributed continuous variables and ordinal variables were summarized as medians with interquartile ranges (Q1, Q3). Comparisons for these variables were conducted using the Mann–Whitney U test for two groups or the Kruskal-Wallis test for multiple groups, while intra-group differences were assessed using the Wilcoxon signed-rank test.

### Efficacy analysis

For the primary endpoint (mRS 0–1 at day 90) and the dichotomized secondary endpoint (mRS 0–2), categorical variables will be compared between groups using the Chi-square test or Fisher’s exact test. To analyze the overall distribution of mRS scores (0–6) as a secondary endpoint, an ordinal shift analysis will be performed using a proportional odds logistic regression model (or the Cochran–Mantel–Haenszel test, adjusted for stratification factors) to evaluate the comprehensive treatment effect across the entire mRS spectrum.

Secondary efficacy outcomes will include NIHSS scores at Day 14, functional independence measured by BI Score at Day 90, and the incidence of stroke recurrence. These outcomes will be analyzed using the Chi-square test. Continuous outcomes, specifically the MoCA and SS-QoL scores, will be compared using either the *t*-test or the Mann–Whitney U test, depending on the data distribution. For repeated measures, TCM Syndrome Scores collected at Days 14 and 90 will be analyzed using a Linear Mixed-effects Model (LMM). This approach will facilitate the assessment of the interaction between group and time.

### Safety analysis

The primary safety outcomes, the incidence of SAEs within 90 days, will be analyzed between groups using Fisher’s exact test. AEs will be compared using the Chi-square test. AEs will be categorized by system organ class and preferred term.

Secondary safety outcomes as Laboratory: Changes in laboratory parameters, including Absolute neutrophil count, platelets, liver and renal function, and coagulation, from baseline to post-treatment will be compared using either the *t*-test or the Wilcoxon signed-rank test. Clinically significant abnormalities in laboratory values findings will be compiled and compared between groups.

### Multiplicity considerations

This study is an exploratory pre-trial designed to assess feasibility, safety, and preliminary signals of effect rather than to confirm multiple efficacy hypotheses. Accordingly, the primary efficacy endpoint was limited to the proportion of patients with 90-day mRS scores of 0–1. All other measures (including NIHSS, BI, MoCA, SS-QoL, stroke recurrence, etc.) were treated as exploratory secondary outcomes.

Because multiple comparisons were performed, there is a theoretical risk of Type I error inflation. However, current methodological consensus indicates that applying strict multiplicity corrections to all secondary endpoints in exploratory trials (for example, Bonferroni adjustment) can substantially raise the risk of Type II errors and obscure potentially important clinical signals. For this reason, no formal multiplicity adjustment was applied in the present study.

We will report *p*-values for all secondary outcomes transparently. Interpretation, however, will emphasize effect size, the 95% confidence interval width, and clinical relevance rather than relying solely on a *p* < 0.05 threshold (21). We emphasize that any positive findings in secondary endpoints are of a hypothetical nature and need to be verified in subsequent confirmatory trials.

### Subgroup analysis

The prespecified subgroup analyses (by age, baseline NIHSS, TOAST classification, and history of comorbidities) aimed to assess whether treatment effects were consistent across patient groups. However, these analyses have important limitations in small-sample pre-trials. First, small subgroup sizes reduce statistical power. Second, repeatedly subdividing the sample increases the risk of selection bias and false positives (22). Although subgroups are pre-specified, subgroup analyses remain exploratory and their findings are generally regarded as of limited reliability, particularly when strong biological rationale is absent. Accordingly, we adopt the following principles. First, all subgroup results are exploratory hypotheses rather than confirmatory evidence. Second, no conclusion about treatment efficacy will be based on the point estimate or *p* value from any single subgroup. Third, a treatment-by-subgroup interaction test will assess effect heterogeneity; however, because of limited sample size, an interaction *p* > 0.05 does not exclude the possibility of true heterogeneity (23); The forest plot will display the OR and 95% CI for each subgroup only when the interaction *p* < 0.10. In that case, the result will be described as a “trend worthy of further verification in subsequent trials” rather than as a statistically significant difference (24). This strategy follows recent methodological recommendations for subgroup analysis: prioritize consistency of effects, avoid overinterpreting subgroup differences from small samples, and reduce misleading inferences by reporting results transparently (22).

### Metabolomics analysis

Inter-group differences were assessed using two-sample *t*-tests conducted in R. *p*-values were adjusted for multiple comparisons through the Benjamini-Hochberg false discovery rate (FDR) method, with significance defined as FDR < 0.05. For confirmatory analyses, the Bonferroni correction was applied. Additionally, fold changes and 95% confidence intervals were reported in conjunction with statistical significance. We applied orthogonal partial least squares discriminant analysis (OPLS-DA) to link metabolic profiles with clinical outcomes. Patients were dichotomized by the primary endpoint—day 90 mRS 0–1 (good functional outcome)—and metabolites from glycolysis, the TCA cycle, and oxidative stress pathways served as predictor variables (X) in the OPLS-DA classification model. For secondary outcomes (for example, the full mRS distribution, NIHSS improvement, MoCA score, and Barthel index), we used partial least squares regression (PLS-R) or multivariable linear and ordered logistic regression, with metabolite profiles as predictors, to assess their joint predictive value for continuous or ordered categorical measures. To evaluate how metabolic features influence the gradient of functional recovery across the mRS distribution, we included scores of the main metabolic components as covariates.

### Missing data

Missing data are inevitable in clinical trials and, if mishandled, can compromise statistical inference and undermine the credibility of results (25). To address this, the study emphasizes prevention by incorporating measures at the trial-design stage: standardizing follow-up procedures, strengthening patient-adherence management, and training the research team to recognize and mitigate missing-data risks.

All missing primary and secondary outcome data will be handled according to the pre-established statistical analysis plan. We will document the specific reasons for missingness (for example, loss to follow-up, death, refusal to assess) and report the proportion and pattern of missing data by group. We will also compare baseline characteristics between participants with and without missing data to evaluate potential selection bias. The primary analysis will use Multiple Imputation (MI) to address missing outcome data. MI is preferred over Complete Case Analysis (CCA) because it preserves all randomized participants, reduces selection bias, and increases statistical power (26). The imputation model will include: treatment allocation; age; sex; baseline NIHSS score; stroke subtype (TOAST classification); enrollment time window; early clinical improvement (e.g., Day 14 NIHSS); occurrence of adverse events; and survival status (patients who die will have mRS assigned a value of 6).

We will generate 50 imputed datasets and combine estimates and their standard errors using Rubin’s rules. To evaluate robustness, we will perform the following sensitivity analyses: (1) complete-case analysis (CCA): include only patients with complete day 90 mRS; (2) worst-case imputation: treat all missing mRS as adverse outcomes (mRS ≥ 3); and (3) death-attribution analysis: separate and explicitly account for deficits attributable to death versus deficits attributable to other causes.

All methods and assumptions for handling missing data will be reported transparently in the final manuscript. This report will include the proportion of missing data, the assumed missing-data mechanism (for example, “Missing at Random,” MAR), and detailed descriptions of the imputation procedures used. We will adhere to current methodological consensus by minimizing omissions, fully characterizing missing data, and applying modern statistical methods to limit the impact of missingness on inference.

## Discussion

The therapeutic potential of YNKC lies in its multi-target pharmacological mechanism. Preclinical evidence suggests that the formulation exerts neuroprotective effects by modulating lipid metabolism, inhibiting oxidized low-density lipoprotein (ox-LDL), vascular endothelial growth factor (VEGF), and intercellular adhesion molecule (ICAM), among other atherosclerosis markers (27). Network pharmacology studies further identified key active ingredients such as saffron acid, quercetin, oleanolic acid, and paeonol (11). Saffron acid can alleviate ischemia–reperfusion injury, while quercetin exhibits significant antioxidant and anti-inflammatory properties (28). Research indicates that quercetin can reduce infarct size, decrease neuronal apoptosis (29), and protect blood–brain barrier integrity and alleviate brain edema by inhibiting the expression of pro-inflammatory cytokines (IL-1β, TNF-*α*, IL-6, IFN-*γ*) (30–32). This study uniquely integrates targeted blood metabolomics analysis to bridge the gap between preclinical findings and clinical phenotypic responses. Ischemic stroke induces a significant systemic energy crisis, marked by disruptions in oxidative phosphorylation and an increase in anaerobic glycolysis. By quantitatively profiling these dynamic metabolic shifts—particularly within the TCA cycle and glucose metabolism pathways—we aim to delineate the systemic regulatory network of YNKC *in vivo*. This objective molecular evidence will clarify how YNKC targets these critical pathological pathways during the acute phase, ultimately reducing infarct volume and enhancing functional recovery.

A defining characteristic of this study is its design as a prospective, randomized, open-label, blinded-endpoint trial with stringent inclusion and exclusion criteria. With a sample size of 80 participants, it is essential to minimize population heterogeneity to maintain high internal validity. We achieve this by selectively enrolling patients within a specific severity range (NIHSS 4–22) and time window (less than 72 h), while also excluding individuals with severe comorbidities. This meticulous approach fosters a homogeneous study population, thereby enhancing our capacity to identify a preliminary therapeutic signal for YNKC. By reducing background noise and confounding variables, this study stands apart from broader, pragmatic trials.

We designated the Day 90 mRS as the primary endpoint, consistent with international standards for stroke research to facilitate comparability (33). Additionally, incorporating TCM syndrome scores alongside the NIHSS enables a thorough assessment. Correlating these multidimensional clinical scales with metabolomic profiles will enable the identification of potential prognostic biomarkers. This integrated approach is particularly well-suited for assessing TCM interventions. It encompasses functional recovery, symptom improvements specific to TCM patterns, and the underlying “multi-component, multi-target” molecular mechanisms.

We recognize several limitations in our study. First, the relatively small sample size (*n* = 80) constrains our ability to definitively confirm efficacy; nonetheless, it is adequate for exploratory analysis aimed at estimating effect sizes and capturing robust metabolic signatures. Second, although the stringent eligibility criteria enhance internal validity, they may limit the generalizability of our findings to the wider, unselected stroke population. Despite these limitations, this study functions as an essential proof-of-concept trial. Positive results will yield the necessary data to inform the design and power of a subsequent large-scale, multi-center Phase III confirmatory trial.

### Protocol amendment

The principal investigator will hold the responsibility for any decisions regarding amendments to the protocol. Should any modifications occur, such as changes to eligibility criteria, outcomes, or analysis, the principal investigator must communicate these changes and obtain approval from the Ethics Committee of Guangdong Provincial Hospital of Chinese Medicine before implementation. Additionally, the principal investigator will be tasked with communicating with other relevant parties.

### Ethics and dissemination

The Ethics Committee of Guangdong Provincial Hospital of Chinese Medicine approved the protocol (BF2025-006-01, V2.0, Apr. 2025). The ethics committee approval document are available in both Chinese and English. The findings of this study may be published in scientific literature; however, the confidentiality of the subjects’ personal information will be preserved in accordance with legal requirements. Personal data will not be disclosed unless mandated by law. Regulatory authorities and the Ethics Committee will have direct access to the subjects’ original medical records for the purpose of verifying clinical trial procedures and data, as permitted by regulations.
